# Ethnobotanical Study of Medicinal Plants of Hezar Mountain Allocated in South East of Iran 

**Published:** 2012

**Authors:** Peyman Rajaei, Neda Mohamadi

**Affiliations:** a *Department of Biology, Faculty of Basic Science, Kerman Branch, Islamic Azad University, Kerman, Iran.*; b *Young Researchers and Elites Club, Kerman Branch, Islamic Azad University, Kerman, Iran. *

**Keywords:** Ethnomedicine, Ethnobotany, Hezar Mt., South East Iran

## Abstract

This manuscript is the result of ethnobotany and ethnopharmacology survey on the Hezar Mountain in SE of Iran. Traditional botanical medicine is the primary mode of healthcare for most of the population of this region. The plants were collected in and around Hezar mountain from 2008-2010. The authors have conducted an interview of total 75 informants; The traditional uses of 92 species belonging to 35 vascular plant families and 78 genera have been recorded. The largest number of medicinal species came from Lamiaceae (15.2%). The most common preparations were decoction and infusion. These species are utilized to treat several ailments which the most common of them are digestive disorders of the gastrointestinal tract, (25.4%), renal and genital disorders (13%), respiratory tract system disorders (11.8%), and heart-blood circulatory system disorders (10.2%) respectively.

## Introduction

There are about 35,000 to 70,000 plant species that have been used for medicinal purposes worldwide (1) from which, the application of 6,500 species is related to the Asia (2). Iran has a long medicinal tradition and traditional learning of plant remedies. One of the earliest ethnobotanical works that we could name here is a work by Hopper and Field (1973) on useful plants and drugs of Iran and Iraq (3). Shokir and Safaian (1993) also presented a list of 210 medicinal plants used in Mazandaran province (4). An ethnobotanical work in Turkmen Sahra area in north of Iran has been done by Ghorbani (2005) (5). In their study, 136 species from 51 families were documented. Miraldi *et al. *reported a total of 30 medicinal plants from the west Azerbaijan villages (6). Naghibi *et al*., represented 46 genera and 410 species and subspecies from Labiatae family in Iran that many members of this family were used in traditional and folk medicine (7, 8). 

The nearest main mountains to Hezar are Lalezar with the highest peak of 4351 m in the West and Jupar with the highest peak of 4135 m in the North. No ethnobotanical study has been reported from these mountains. Hezar is a high mountain and there are many villages around it, far from the cities, so that their people have a little access to medical facilities. This fact encourages local people to use herbal plants for the treatment of some daily diseases. Due to the fallowing reasons, this mountain has been chosen as an ethnomedicinal case study in the present survey: (a) Hezar mountain has a rich plant diversity, (b) Owing to the height of this mountain (4465 m), Hezar has not been adequately explored, (c) The rich traditional knowledge of the native habitants regarding different plant species, handed down from their predecessors.

The aim of this paper is to focus on the kinds of medical diversity found in the study area, depending on the frequency of the plants medical applications, and to show the most common preparations mode of botanical drugs used in Hezar ethnomedicine.

**Figure 1:**
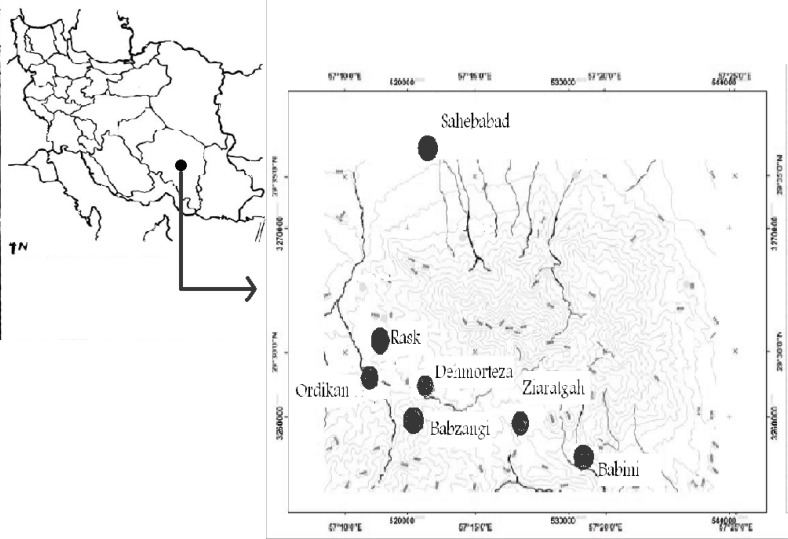
Study area map in SE of Iran

The area under the study, Hezar (29°30/ N, 57°20/ E) with an area of 900 Km2 and height of 4465 m, is the 4th highest mountain allocated in the south east of Iran (Figure 1).

*Climate and vegetation overview*

**Figure 2:**
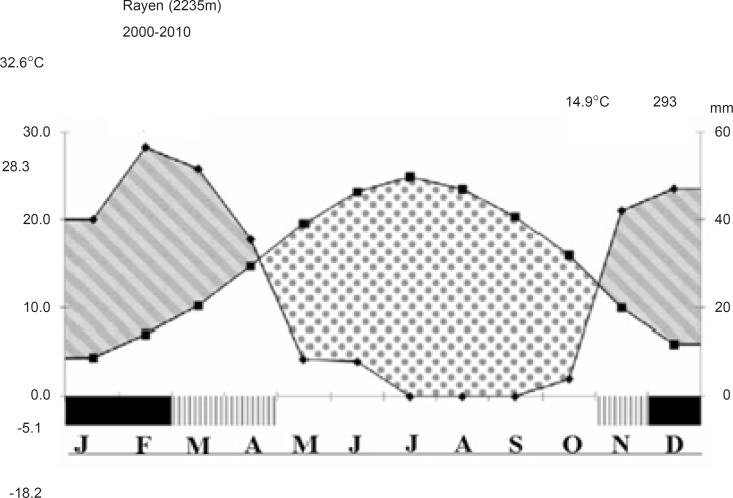
Ecological climate diagram of Rayen meteorological station, indicating monthly average of temperature and precipitation and relative humidity (hatched) and relative arid (stippled) seasons

The climatic information was obtained from the nearest meteorological station-Rayen, the station located at 15 Km NE of study area at 2235 m altitude. The climate of this area is arid and cold. The lowest and highest absolute temperatures are -1°C and 40°C. In most parts of Hezar, the precipitation is increased by altitude. The main period of precipitation is during the late of autumn, winter and early spring. The data of Rayen station at the NW of Hezar, shows that the winter, spring, autumn and summer rainfall comprises 56%, 22%, 32% and 1% of the annual precipitation, respectively. The average number of frost days is 73 with a maximum during the January. Ecological climate diagram shows that the period of aridity of this area starts from late April to the end of November. The period of humidity is pronounced to November during up to early April (Figure 2).

The results of this study show the existence of five different habitats in the studied area as follows: 

I. Plain regions: They include all mountain slopes and the flat plains between the mountains reaching areas mildly steeped. Being a region fit for agricultural activity, various species belonging to Poaceae and Apiaceae distributed in this region: *Conium maculatum, Levisticum officinale, Dorema aucheri*, *Dorema ammoniacum*, *Artemisia aucheri*, *Cirsium arvense*, *Cousinia onopordioides*, *Echinops lalesarensis, Hertia intermedia, Scorzonera mucida*, *Scorzonera pussila, Tanacetum parthenium*, *Berberis integerrima *and *Lonicera nummulariifolia*. 

II. Rocky areas: These habitats are usually situated on the slopes strew with rocks and boulders.

These areas are covered by species such as *Corydalis rupestris, Graellsia saxifragifolia, Psychrogeton *spp., *Arnebia euchroma, Rosularia modesta*, *Melica persica *and *Parietaria judaica*. 

III. Screes are the major habitats of the alpine zone in Hezar Mountain. *Cicer tragacanthoides, Asperula glomerata, Astragalus tenuiscapus, Silene daenensis *and *Nepeta natanzensis *are the important species of Hezar scree habitats. The species richness of these habitats is very low. 

IV. Humid mountainous regions: In this habitat, various grass species and some other families such as the followings are found: *Artemisia tournefortiana, Primula capitellata, Carex songorica, Glaux maritima, Orchis coriophora, Juncus inflexus, Taraxacum spp. *and *Veronica anagallis-aquatica*.

V. Degraded areas: These habitats include manipulated rural areas used for farming and gardening as well as diggings alongside the roads and pathways. They are local to various grasses and weedy species such as: *Malcolmia africana, Malva neglecta, Cynodon dactylon, Geranium tuberosum, *and *Cardaria draba*.

## Experimental

*Data collection*

The different slopes of Hezar were investigated and the specimens were collected by authors during 2008-2010 between March and October in each year. Then, ethnobotanical information have been gathered from the main villages in and around of Hezar Mountain. These villages have been selected based on their population including Babini (2600 m), Ziarat-gah (3000 m), Babzangi (3300 m), Ordikan (3200 m), Rask (3000 m), Deh-morteza (2950 m) and Sahib-Abad (2500 m) (Figure 1). The specimens were prepared according to the established herbarium techniques. The plant samples were recognized according to 

Flora Iranica (9), Flora of Iran (10), Flora of Iraq (11), and Flora of Turkey (12) and kept in the Avicenna Herbarium of Islamic Azad University, Science and Research branch, Tehran, Iran (IAUH).

*Interviews with local people*

The data were collected through direct interviews. A questionnaire was administered to the local people (Appendix 1). After collecting the specimens, we showed these fresh specimens to the local people in their houses and farms. We employed random sampling techniques to identify potential participants and interviewed a total of 75 people (30 men and 45 women) whose age ranged from 40 to 75 years. The women were said to have better knowledge about the medicinal application of plants than men. Most of the interviewees mainly belonged to families which still have a strong connection with traditional agricultural and pastoral activities. There were herbalists, healers and plant traders among the interviewees as well. The same plant specimens were shown to different people to confirm the accuracy of the results. The interviews consisted of obtaining the information related to the identification of plants, their vernacular names, their medical uses and the preparation of remedies.

## Results

*Medicinal plants*

A total of 92 species belonging to 35 vascular plant families and 78 genera were reported. From these species, 79 species reported to be used for medicinal applications, 70 species of which were used to treat more than one disease and the remaining 9 species were used to treat only one disease.

The largest number of medicinal species came from Lamiaceae (15.2%), followed by Asteraceae (12%), Fabaceae (8.7%), Apiaceae (8.7%), Rosaceae (5.4%) and Brassicaceae (5.4%). Recorded plant species with their vernacular names, uses and mode of preparation are listed in Table 1.

**Table 1:** List of medicinal plant species and medical health data

**Scientific name**	**Local name**	**Part used**					
**Apiaceae**							
***Buplereum falcatum ***L.		L, Se					
***Conium maculatum ***L.	kama						
***Ducrosia anethifolia ***(DC.) Boiss	Gicho	L					
***Dorema ammoniacum ***D. Don.	Anghuzeh	La					
***Dorema aucheri ***Boiss.	Kal	La					
***Ferula hezar- lalehzarica ***y. Ajani	Kahoo vahshi	St, Rh					
***Ferulago carduchorum ***Boiss. and Hausskn***.***	Garchik	La					
***Levisticum officinale ***W. D. Koch.	Karafse kuhi	L, R, Se,					
**Asteraceae**							
***Achillea wilhelmsii ***C.Koch.	Bumadarun	L					
***Arctium lappa ***L.	Baba adam	R, L					
***Artemisia aucheri ***Boiss.	Dormane	L					
***Artemisia persica ***Boiss.	Dormane turky	Fl, L	IT	25	Fever, Gastric disorder	Hydrodistilation	508
***Cichorium intybus ***L.	Kasni	Wh.p	PL	30	Jaundice, Blood sugar	Hydrodistilation, Decoction	512
***Cirsium arvense ***(L.) Scop.		R	PL	3	Dermal inflation, appetizing	Poultice	513
***Hertia intermedia ***(Boiss.) O. Kuntze.		L	IT-SS	2	Dermal inflation, insects sting	Poultice	523
***Launea acanthodes ***(Boiss.) O. Auntze.	Goja	L, St	IT	7	snake bites, insect sting, Antiseptic	Poultice in form of ash	526
***Scariola orientalis ***(Boiss.) Sojak.			IT	4	Use by bees as food		531
***Tanacetum fisherea ***Aitch. and Hemsl.	Chaye ahshi	L	IT	6	Nerve system relaxant	Decoction	537
***Tanacetum parthenium ***(L.)	Babune gavi	L	PL	12	Fever, Sedative, Gastric disorders, Nerve system relaxant	Decoction	538
Berberidaceae							
***Berberis integerrima ***Bge	zarch	Fr, R	IT	36	Blood Sugar and Depurative	Decoction, Edible	543
Boraginaceae							
***Arnebia euchroma ***(Royle.) l. M. Johnst.	havachue	R, Se	IT	48	Wound, Cutting	Poultice	544
***Asperugo procumbens ***L.		Fl	IT-M	2	Nerve system relaxant, antispasmodic, Menstruate	Decoction	545
Brassicaceae							
***Capsella bursa- pastoris ***(L.)	Kise keshish	A.p	Cosm	18	Coagulation, antiepileptic. Hypertension, Diarrhea	Infusion	558
***Cardaria draba ***(L.) Desv.	Mocoo	Wh.p, Se	IT-M-ES	39	Diuretic	Decoction, Edible	559
***Descurainia Sophia ***(L.) chur.	Khakshir	Se	IT-M-ES	48	Asthma, Stomachache, Heatstroke, Lِaxative, Appetizing	syrup	564
***Eruca sativa ***Lam.	Mandab	Se	PL	25	Hair loss, mange, Making oil	Oral, Poultice	567
***Erysimum crassicaule ***(Boiss.) Boiss.		Fl	IT	4	Respiratory disorder, Digestive spasm	Infusion	568
**Caryophyllaceae**							
***Acanthophyllum laxiusculum ***Schiman- Czeika.	Choobe	R	IT	3	use as a kind of food(Halva), detergent	Edible	581
Chenopodiaceae							
***Chenopodium foliosum ***(Moench) Aschers ***.***	Khorma	L	PL	12	Diuretic	Decoction	600
**Convulvolaceae**							
***Convolvulus arvensis ***L.	Pichak	Wh.p	PL	3	Laxative, Carminative	Decoction	607
***Convolvulus spinosus ***Burm.	Pichak	R	IT-SS	2	Fever, Purgative	Decoction	608
**Cucurbitaceae**							
***Bryonia aspera ***Stev. Ex Ledeb.	Angoor vahshi	Angoor vahshiWh.p	IT	15	Digestive problems for domestic animals, Liver problems	Oral	611
**Cupressaceae**							
***Juniperus excelsa ***M. B.	Ors	Fr	IT-ES	18	Diuretic, Rheumatism, antiseptic,Dermal inflations, Boils	Infusion	
**Ephedraceae**	Khimook	L, Sh	IT-ES	35	Diuretic, Gastric and intestine inflation, Diaphoretic	Decoction	616
***Ephedr***a ***major ***Host.	Afkoo	La	IT	-	Poisonous		
**Euphorbiaceae**							
***Euphorbia ***spp.** Fabaceae**							
***Astragalus*** ***camptoceras ***Bunge.	Kalilolmolk	Se	IT	31	Cold	Decoction	624
***Astragalus dschuparensis ***Freyn. and Bornm.	Gini	La	IT	9	Body reinforce, Making tragacanth use as detergent, Produce rope	Bath, use by bees as food	628
***Cercis siliquastrum ***L.	Arghavan	Ba, L	IT-ES	3	Constipation	Decoction, Edible	
***Glycyrrhiza glabra ***L.	Matki	R. Rh	IT-M-ES	39	gastric ulcer, Laxative, Bronchitis	Decoction	
***Lathyrus sativus ***L.	Karoo	Wh.p, Fr	PL	28	Diarrhea, Carminative	Infusion, as a kind of soup	643
***Medicago sativa ***L.	Yonjeh	A.p	IT	40	Blood fat	Infusion	644
***Melilotus*** ***officinalis ***(L.) Desr.	Yonjeh zard	Fl, A.p	IT-M-ES	13	Headache, diuretic, Nerve system relaxant	Infusion	646
***Robinia pseudoacacia ***L.	Aghaghia	Fl, L	ES	3	Purgative, Diuretic, Laxative	Decoction	649
**Fumariaceae**							
***Fumaria asepala ***Boiss.	Shatareh	L	PL	25	Carminative, Stomachache, Depurative, Fever,	Decoction	652
**Juglandaceae**							
***Juglans regia ***L.	Ghoz	L, B. Fr	IT-ES	36	Blood sugar, Bone ache, hair color	Infusion, Edible	
**Gentianaceae**							
***Centurium pulchellum ***(Swartz.) Druce.		La	IT-M-ES	2	Fever, Gall stone	Oral	
**Geraniaceae**							
***Bieberstainia multifida ***DC.*	Bahman	R, Tu	IT-M-ES	35	Carminative, Backache, Sedative, Sexual instinct	Decoction	653
***Erodium cicutarium***(L.) ’Her		Wh.p	IT-M-ES	14	coagulation, Constipation	Decoction	654
**Juncaceae**							
***Juncus inflexus ***L.		A.p	PL	4	Making rope and basket		658
**Lamiaceae**							
***Dracocephalum*** ***polychaetum ***Bornm.	Zaroo	L, Fl	IT	65	Carminative, Rheumatism, flavor in whey	Decoction, Hydrodistilation	659
***Marrubium*** ***anisodon ***C. Koch.		A.p	IT-ES	15	Fever, Hypertension, cardiac pains, Menstruate	Infusion, Oral	662
***Marrubium vulgar ***L.		A.p	IT-M	16	Diuretic, Appetizing	Infusion, Oral	663
***Mentha*** ***longifolia ***(L.) Hudson.	Poodeneh	A.p	IT-M	48	Fever, Sedative	Infusion	
***Nepeta bracteata ***Benth.	Zufa	Fl	IT	42	Cold, Cough, Expectorant, Constipation, infertility	Decoction	665
***Nepeta glomerolosa ***Boiss.		Fl	IT	25	Dermal inflation, pneumonia	Decoction	669
***Nepeta ispahanica ***Boiss.		Fl	IT	28	Respiratory disorder, Gastric and intestine disorder	Decoction	670
***Nepeta saccharata ***Bunge.	Badranj	L, Fr	IT	31	Cold, Backache	Decoction	675
***Salvia nemerosa ***L.		Fl	IT-ES	8	Use by bees as food	Use by bees as food	677
***Salvia sclarea ***L.		L,Fl,Se	IT-SS	12	Kidney stone, its extract use as perfume	Infusion	
***Teucrium polium ***L.	Kalpoore	A.p	PL	42	Stomachache, Carminative	Hydrodistilation, Oral	
***Thymus carmanicus ***Jalas.	Apishan	L, Fl	IT-M	65	Cold, Sedative, Asthma, Diarrhea	Decoction	680
***Ziziphora clinopodioides ***Lam.	Aghlale	L, Fr	IT	70	Nerve system relaxant	Infusion, Decoction	681
***Ziziphora tenuir ***L.	Kakuti	L	IT	54	Nerve system relaxant	Infusion	682
**Liliaceae**							
***Colchichum schimperi ***Janka.	Gole hasrat	B	IT	5	Backache	Poultice	683
***Eremurus persicus ***(Jaub. and Spach) Boiss.	Hasan aloo	L	IT	23	Use as a kind of food, Diuretic	Edible	684
***Tulipa biflora ***Pall.	Lale	B	IT	23	Use as a kind of food	Edible	689
**Onagraceae**							
***Epilobium minutiflorum ***Hausskn.	Pudene sagi	Rh, Fl	IT	6	Constipation	Decoction, Oral	693
**Orchidaceae**							
***Orchis coriophora ***L.		Tu	IT-ES	2	Cold, Cough, Diarrhea	Decoction	695
***Malvaceae***							
***Althaea officinalis ***L.	Khatmi	L, Fl	ES	19	Diuretic, Laxative, Cold, Sore throat	Decoction, Infusion	691
***Malva neglecta ***Willr.	Panirak	A.p	IT-M-ES	25	Inflation, Sedative	Decoction	692
**Papaveraceae**							
***Papaver dubium ***L.	Shaghayegh		IT-SS	27	Poisonous		700
**Plantaginaceae**							
***Plantago lanceolata ***L.	Kochak	L, R, Se	IT-M-ES	12	Jaundice, Sedative	Decoction, Poultice	703
**Polygonaceae**							
***Rheum ribes ***L***.***	Rivas	L, Fr, Fl, Se	IT	34	Use as a kind of food, Laxative, Making jam, Hair dyeing, Anti parasitic worn in domestic animals	Edible, infusion	729
***Rumex crispus ***L.	torshak	R, Rh	IT	14	Fever, Diarrhea, Laxative	Decoction	730
***Rumex dentatus ***L.	torshak	L	IT	12	Vegetable for a kind of food	Edible	731
Ranunculaceae							
***Adonis aestivalis ***L.	Teryakoo	Fl	IT-M-ES	25	Kidney stone, Laxative, Nerve system relaxant, Diuretic	Decoction	734
***Consolida orientalis*** *)*Gay)Schrod.		Fl, L	IT-M	5	Kidney stone, Hair loss	Poultice, Decoction	737
Rosaceae							
***Amygdalus eburnea ***Spach.	Ghoos	Se	IT	18	Making oil	Edible	742
***Amygdalus elaeagnifolia ***Spach.	Archan	Se	IT	16	Making oil, Dye	Edible	743
***Cotoneaster nummularia ***Fisch. and C. A. Mey.	Shir khesht	Wh. p	IT	6	Fever, Laxative, Jaundice	Decoction	744
***Rosa beggeriana ***Schrenk	Korik	Fl	IT	25	Cardiac disorders	Hydrodistilation	747
***Sanguisorba minor ***Scop	Gheitaran	L, St	IT-M-ES	36	Toothache	Decoction	748
**Rubiaceae**							
***Galium aparine ***L.		A. p	PL	3	Kidney stone, Diuretic	Infusion	751
**Salicaceae**							
***Salix alba ***L.	Bid	Ba	IT-M-ES	32	Fever, Jaundice	Decoction, Oral	753
**Scrophulariaceae**							
***Scrophularia leucoclada ***Bunge	Mokhalaseh	L, Se	IT	41	Bronchitis, apoplexy	Oral	757
***Verbascum carmanicum ***(Bornm.) Hub.- Mor.	Gole mahoor		IT	6	Sedative, Diuretic, respiratory disorder	Infusion	759
**Solanaceae**							
Hyoscyamus spp			IT	-	Poisonous		
Hyoscyamus spp							
***Daphne oleoides ***Schrev	Terbid	Fr, R	IT	13	Purgative, Laxative, Making jam	Oral	772
Urticaceae	Gazane	R, A.p	PL	35	Coagulation, Blood sugar, foot pain	Decoction, Poultice	774
***Urtica dioica ***L. ssp. ***dioica***							
Zygophyllaceae							
***Peganum harmala ***L.	Espand	L, Se	IT-M-ES	28	Noise blooding, Antiseptic, insomnia	Poultice, smoke	776
***Tribulus trrestris ***L.	Kharkhasak	Fr	PL	34	Kidney stone, Diuretic	Infusion	777

R: Root; St: Stem; L: Leaf; Fl: Flower; Fr: Fruit; Se: Seed; A. p: Aerial parts; Wh. p: Whole plant; Tu: Tuber; B: Bulb; Ba: Bark; Rh:Rhizome; La: latex

**Figure 3:**
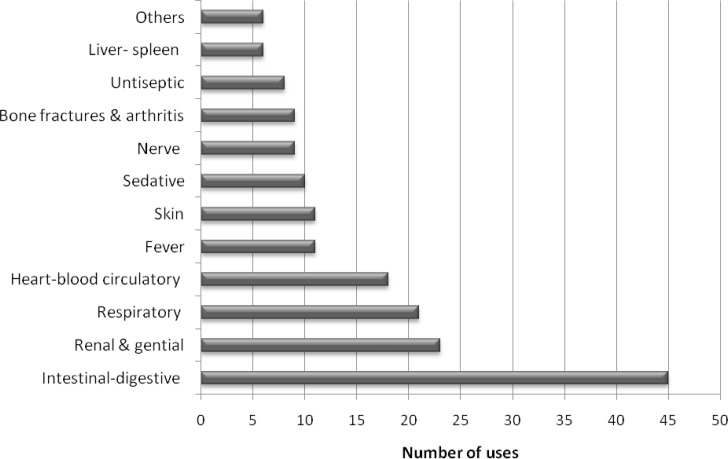
Remedies reported by informants

As a result, we observed that these plants are used especially for intestinal digestive disorders of the gastrointestinal tract, (25.4%), renal and genital disorders (13%), respiratory tract system disorders (11.8%), heart-blood circulatory system disorders (10.2%), fever (6.2%), skin disorders (6.2%), sedative (5.6%), bone fractures and arthritis (5%), nerve disorders (5%), antiseptic (4.5%), liver-spleen disorders (3.4%) and others (nose bleeding, body reinforcement, appetizing, *etc.*) (3.4%) (Figure 3).

**Figure 4:**
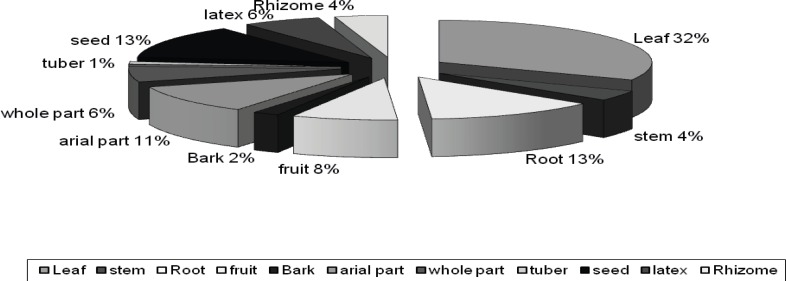
Plant parts use and their percentages

**Figure 5:**
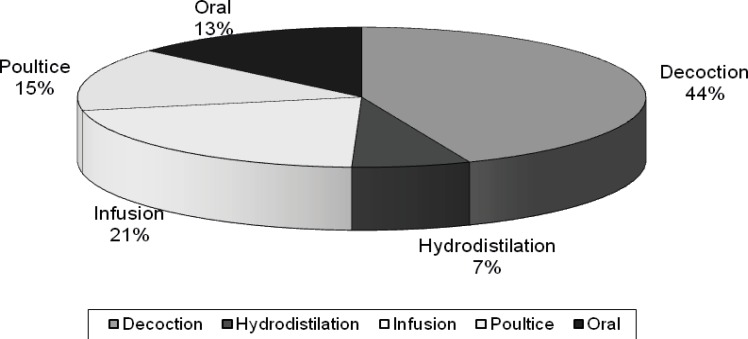
Mode of preparations and their percentages

Our results showed that several parts of individual plant species are used as medicine. The most widely used medicinal plant part was the leaves (33 species) followed by flowers (18 species), root (14 species), Seed (14 species), aerial parts (10 species), fruit (8 species), whole plant (7 species), latex and seed (6 species), rhizome (4 species), stem (4 species), bulb (3 species), tuber (2 species) and bark (2 species) (Figure 4). The most common mode of preparation was decoction (44%) and followed by infusion (21%), poultice (15%), oral (13%) and Hydrodistillation (7%) (Figure 5).

The study of the growth form of the medicinal plants revealed that herbs made up the highest proportion of medicinal plants (80 species), followed by shrubs (7 species) and trees (5 species).

**Figure 6:**
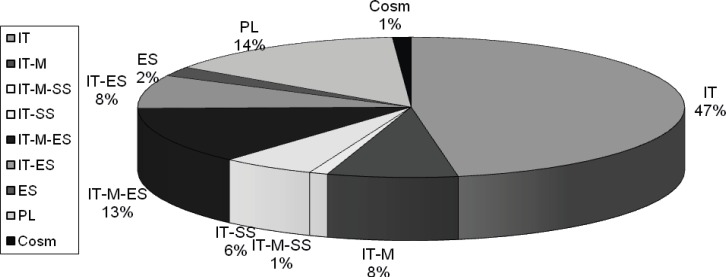
Chorology of plants and their percentages (IT: Irano-Turanian; Cosm: Cosmopolitan; ES: Euro-Siberian; PL: Pluriregional; M: Mediterranean; SI: Sahara-Indian).

*Chorology: *Irano-Turanian elements compose 48% of the medicinal plants growing on the study area (Zohary, 1973). They are the dominant chorology in the region (Figure 6).

*Plants used in veterinary*

Some species are used in veterinary. The species include root of *Bryonia aspera *Stev. Ex Ledeb is used for digestive problems in sheep and horses. The oil obtained from the seeds of *Eruca sativa *L. was used for the remedy of mange and dermal problems in domestic animals. Fruit of *Rheum ribes *L. was used as antiparasitic worm in domestic animals*. *Ash of leaves and stem of *Launea acanthodes *(Boiss.) O. Auntze which are used for snake bites and insect sting.

*Plants with non-medicinal uses*

Seventy species have been recorded for non-medicinal uses including, edible, natural dyes, making gum, rope, basket, ink and detergent. Root of *Acanthophyllum laxiusculum *Schiman-Czeika is used for a kind of food (Halva) and detergent. *Dracocephalum polychaetum *Bornm and *Levisticum officinale *W. D. Koch are used as flavor in yoghurt. *Juncus inflexus *L. is used for making rope and basket. *Juglans regia *L. and *Berberis integerrima *Bge are used in dyeing. *Daphne oleoides *Schrev is used for making ink. *Ephedr*a *major *Host is used for making Mashk (a bag made from animal leather and used for maintaining water and yoghurt). The bulbs of *Tulipa biflora *Pall leaves of *Rheum ribes *L. and *Rumex dentatus *L. are used for local foods. The latex of *Dorema aucheri *Boiss, which is called Eshterk, is used for Making Gum. The latex of *Astragalus dschuparensis *Freyn and Bornm., which is a kind of detergent.

## Discussion

As a result of the rich flora of this region, medicinal plants are the most important means of health care. Due to the lack of modern medicine, difficult geography of the district as well as traditional culture cured by plants, serves as a usual way in this region particularly in elders.

Some species are used so frequently that are mentioned by every interviewee. Our results indicated that medicinal species such as *Levisticum officinale*, Artemisia persica, Thymus carmanicus and Ziziphora clinopodioides are mentioned by many informants. *Arnebia euchroma *(Royle) Jonst (Boraginaceae) is a perennial plant of the alpine region, distributed in the Persia, Pamir, Tien- Shan, Himalaya and the western Tibet, with an altitudinal range between 3700 and 4200 m above the sea level. Local people of Hezar Mt., use the roots of *A. euchroma *as poultice for wound healing. Shikonin and its derivatives extracted from the roots of *A. euchroma *have been known since ancient times and used as dyes for silk and food products. Shikonin possesses antibacterial, antifungal and anti-inflammatory properties. Furthermore, *A. euchroma *exhibits potent anti-HIV activity (13). Arnebin-1 and arnebin-3 derived from it possess anticancerous properties (13). Due to its medicinal uses, the species is being harvested indiscriminately from the wild both for domestic and Pharmaceutical purposes. This has resulted in *A. euchroma *critically endangered the status and its listing in the species prioritized for conservation in this region.

*Levisticum officinale *W. D. Koch (Umbellifera) is a perennial plant of the alpine region distributed only in the SE of Iran (Hezar Mt.) and E of Afghanistan. The essential oil of *L. officinale *was characterized by large amount of monoterpenes, approximately 93.8%. *L. officinale *exhibits various pharmacological and biological activities, including estrogenic (14), apoptotic (15) and antimycobacterial (16) activities.

The main components in the oil were *β*-phellandrene and *α*-terpineol (17). It is a warming and tonic herb for the digestive and respiratory systems. It is used primarily in the treatment of indigestion, poor appetite, wind, colic and bronchitis. The roots, leaves and fruits are antispasmodic, aromatic, carminative, diaphoretic, digestive, diuretic, mildly expectorant and stimulant. Raw or cooked leaves and stems are used as savory flavoring in salads, soups, stews, *etc*. In addition, some species have other uses which food is the most important one such as *Dracocephalum polychaetum *as a flavor in whey. The flavones like Levoteolin and Apigenin are the most components in *Dracocephalum polychaetum.*

Another well-known identified medicinal substance in this mountainous region is Mumenaei. This traditional drug is widely distributed in Russia (termed there Mumie or Mumiyo, India (Saljit), Birma [Kao-tun (blood of the mountain)], Altai Mountains [Barachgschin (oil of the mountain)] Mongolia [Brogschaun (mountain juice)] and Iran. It is found at high altitudes as deposits in walls and caves where they are embedded into rocks. Mumenaei has been used as a medicine by the local people for a long time for bone fracture relief, muscular pain, and arthritis. It is used as a poultice in fracture limb or is eaten orally for the relief of pain. The main organic components, the wax esters and the glycerol ethers, are known to display neuroprotective potential. Future studies will prove whether the monoalkyl ethers also display anti-stomach ache capacity. Finally, the triglycerides have to be studied for their putative antimicrobial activity. The inorganic component(s), the minerals existing in Mumenaei, may have their ameliorating function in bone diseases (18).

New research on some plants of this area such as *Nepeta assurgens *and *Thymus carmanicus *(Labiateae) showed that methanolic extracts of these plants had antibacterial activity and can be used in different cases of bacterial infections especially nosocomial infections (18). Leaves and flowers extracts of *Salvia rhytidae *showed antibacterial activity against *Staphylococcus aureus, Escherichia coli and Pseudomonas aeruginosa*. *S. aureus was *more sensitive to other bacteria. It can be exploited and applied to food system protection, and treatment of bacterial infections (19). In spite of these findings, no ethnobotanical applications were mentioned for *Salvia rhytidea *and *Nepeta assurgens *by informants. Considering the lack of information about the uses and effects of medications of some species like *Dorema ammoniacum*, *Levisticum officinale*, *Artemisia persica*, *Tanacetum fisherea*, *Arnebia euchroma*, *Tribulus terrestris *and *Dracocephalum polychaetum *will be proposed for further examination.

These plants are used in the treatment of some very common ailments like diarrhea, stomach problems, blood sugar, bronchitis and asthma. All diseases might be related to poor hygienic conditions with regard to food and water.

The data of Table 2 shows ethnobotanical comparison between Turkmen Sahra (NE Iran) (5) and Hezar Mt., (SE Iran).

**Table 2:** The comparison with NE Iran (Turkmen Sahra).

		**In SE Iran (Our study)**	**In NE Iran (5)**
Number of taxa	Family	35	41
	Genera	87	72
	Species	92	136
Predominant families Common mode of preparation Part Predominant families used Most reported medicinal uses Dominant chorology		Lamiaceae (15.2%)	Asteraceae (10.9%)
		Asteraceae (12%)	Lamiaceae (9.8%)
		Apiaceae (8.7%)	Apiaceae (8.7%)
		Fabaceae (8.7%)	
		Decoction-Infusion	Decoction-Demulcent
		Leaf (32%)	Leaf (22%)
		Root (13%)	Fruit (12%)
		Seed (13%)	Seed (12%)
		Aerial parts (11%)	Aerial parts (12%)
		Gastrointestinal	Gastrointestinal
		Renal and genital	Skin
		Respiratory	Cardio-Vascular
		Cardio-Vascular	Renal and genital
		Irano-Turanian (48%)	Irano-Turanian (35%)

*Medicinal plants threats*

In recent years, aridity and low precipitation have damaged the vegetation of plants in Hezar. In addition, overgrazing impact is increasingly threatening the fragile medicinal plants in this mountain.

Some rare species such as *Levisticum officinale, Thymus carmanicus, Arnebia euchroma, Dracocephalum polychaetum *and *Dorema ammoniacum *have been threatened as herbalists and traders hire the local people for gathering these species due to the economic purposes. Many of these plants are potentially endangered and vulnerable taxa. Since the alpine plants grow very slowly, they cannot quickly re-grow leaves or flowers that are lost. Harvesting of roots, bulbs, seeds and flowers, which are essential to the survival of the plants, often lead to vanish this species. In addition, local people sometimes sell these medicinal plants in the local market for their livelihood. So, the domestication of these plants is a need for conservation.

Lately, a Manganese purification factory has been established in 30 Km of NW of Hezar Mountain, so that the air pollution and soil toxicity are inevitable in this region. It is a direct threat to flora and fauna in the study area.
